# Allosteric sodium binding cavity in GPR3: a novel player in modulation of Aβ production

**DOI:** 10.1038/s41598-018-29475-7

**Published:** 2018-07-23

**Authors:** Stefano Capaldi, Eda Suku, Martina Antolini, Mattia Di Giacobbe, Alejandro Giorgetti, Mario Buffelli

**Affiliations:** 1 Department of Biotechnology-University of Verona, Verona, Italy; 2 Department of Neurosciences, Biomedicine and Movement Sciences-University of Verona, Verona, Italy; 3 Computational Biomedicine, Institute for Advanced Simulation IAS-5 and Institute of Neuroscience and Medicine INM-9, Forschungszentrum Jülich, Germany

## Abstract

The orphan G-protein coupled receptor 3 (GPR3) belongs to class A G-protein coupled receptors (GPCRs) and is highly expressed in central nervous system neurons. Among other functions, it is likely associated with neuron differentiation and maturation. Recently, GPR3 has also been linked to the production of Aβ peptides in neurons. Unfortunately, the lack of experimental structural information for this receptor hampers a deep characterization of its function. Here, using an *in-silico* and *in-vitro* combined approach, we describe, for the first time, structural characteristics of GPR3 receptor underlying its function: the agonist binding site and the allosteric sodium binding cavity. We identified and validated by alanine-scanning mutagenesis the role of three functionally relevant residues: Cys267^6.55^, Phe120^3.36^ and Asp^2.50^. The latter, when mutated into alanine, completely abolished the constitutive and agonist-stimulated adenylate cyclase activity of GPR3 receptor by disrupting its sodium binding cavity. Interestingly, this is correlated with a decrease in Aβ production in a model cell line. Taken together, these results suggest an important role of the allosteric sodium binding site for GPR3 activity and open a possible avenue for the modulation of Aβ production in the Alzheimer’s Disease.

## Introduction

G-protein coupled receptors (GPCRs) are the largest and the most heterogeneous group of proteins in the eukaryotic genome^1,2^. They consist of seven transmembrane alpha-helices that span the entire width of the membrane with the N-terminus positioned outside the cell and the C-terminus located in the cytosol. These receptors receive messages from the extracellular environment and, through conformational changes, they codify and transmit these messages inside the cell, activating several intracellular pathways. These activations affect the production of hundreds of second messenger molecules as Ca^+^ and cyclic AMP (cAMP)^3^.

The life-cycle of a GPCR can be summarized in four states: ligand binding, G protein coupling, receptor desensitization through the interaction with β-arrestins, and receptor recycling^4,5^. Since this process is very complex and involves a tremendous variety of signaling molecules, GPCRs are the target of a large number of pharmaceutical compounds. In fact, between 20–30% of FDA-approved medications target GPCRs^6^. The most prominent therapeutic applications involving GPCRs include opioid analgesics, antihistamines, anticholinergics, typical and atypical antipsychotics, antimigraine drugs, b2-agonists for asthma, and anti-hypertensives^7^.

Misregulation of GPCRs activity is known to be implicated in the onset and progression of many different pathologies, including many types of cancers, cardiovascular diseases, metabolic disorders, and neurodegenerative diseases^8,9^. Among these, Alzheimer’s disease (AD) has the most increasing trend in incidence and mortality. AD is a progressive neurodegenerative disorder and, accounting for 50 to 70% of the cases, it is the most common form of dementia^10,11^. It is estimated to affect more than 45 million people worldwide (5.5 million only in the US) as a result of global population aging^12–14^. The early symptoms of AD usually appear in the sixth decade of life and include cognitive deficit, apathy, and short-term memory loss^15–18^. Although the etiology of AD is still unclear, the accumulation and deposition of amyloid beta peptide (Aβ) fibrils in the intracellular and extracellular space are recognized as key events for the neuronal damage and synaptic failure in AD patients^19,20^. The two most abundant amyloidogenic Aβ peptides (1–40 and 1–42) are generated from the sequential cleavage of the large Amyloid-β precursor Protein (APP) by β-secretase (or BACE1) and γ-secretase complex^21–25^. The β-secretases and γ-secretases play a fundamental role in amyloid precursor protein (APP) proteolysis and Aβ generation and are therefore regarded as the two major targets in AD drug discovery.

Recently, a high-throughput functional genomics screening, identified the G-protein coupled receptor 3 (GPR3) as a potent modulator of the APP processing^26^. GPR3, an orphan class A GPCR, is highly expressed in central nervous system (CNS) neurons, where it was associated to neuron differentiation and maturation^27,28^, and in ovary and testes, where it is involved in the maintenance of meiotic arrest in oocytes^29^. GPR3 acts as constitutive activator of adenylyl cyclase^30,31^ and its physiological ligand, if exists, is still unknown. Several endogenous molecules, including sphingosine 1-phosphate (SP1) and dihydrosphingosine 1-phosphate (DHSP1), have been proposed as potential ligands for GPR3 but with contrasting results^32,33^. More recently a synthetic molecule, diphenyleneiodonium chloride (DPI), has been reported to act as a GPR3 agonist, increasing the GPR3-stimulated cAMP production and the receptor desensitization and internalization^34^. Overexpression of GPR3 in APP-processing cells significantly increases the production of both Aβ 1–40 and Aβ 1–42 by enhancing γ-secretase activity with a mechanism that promotes the assembly and the trafficking of its components to the cell membrane. Moreover, GPR3-deficient mice exhibit lower accumulation of Aβ peptides^26^. In a subsequent work, the effect of the receptor on Aβ production has been ascribed to its ability to directly interact with APP, and the formation of this complex seems to be mediated by the recruitment and binding of β-arrestin 2 (βarr2) to GPR3^35^. All these studies, however, are devoid of a complete structural analysis of the binding cavity, giving only experimental information on ligands affinity.

Recent progress in GPCRs crystallography opened an unprecedented avenue for receptor-ligand characterization. Nevertheless, the lack of structural data for about the 95% of the members of the family^36^, including GPR3, calls upon the development of specific pipelines in which, GPCRs-targeting *in silico* tools are combined with extensive *in vitro* experiments.

Using computational tools, we were able to model the structure of the GPR3 and perform docking and molecular dynamics simulations with its known agonist, DPI. Based on the computational predictions, we identified three key residues and, together with our *in vitro* experiments, described the effect of their mutations on constitutive and ligand-stimulated activity of the receptor and on the GPR3-induced production of Aβ in model cell line. One of these mutants turned out to completely abolish the signal of the receptor, acting as an inverse agonist, with the consequence of decreasing the production of Aβ. Thus, our results could provide an initial starting point for a specific drug design pipeline of the GPR3 receptor.

## Results and Discussion

### Homology modeling and molecular dynamics simulations

GPR3 is a fascinating GPCR receptor both from a structural and functional point of view. It is known to be involved in many molecular pathways, from modulating the early phases of cocaine reinforcement^37^ to the maintenance of meiotic arrest in rodent oocytes^30^ and emotional-like responses^38^. Very recently it was discovered to play a fundamental role in modulating the amyloid-beta peptide generation in neurons through the interaction with βarr2^26^. The finding that certain G protein–coupled receptors (GPCRs), including also the β2-adrenergic receptor in addition to the GPR3, can regulate Aβ production^39^ has offered new avenues for Alzheimer’s drug discovery. In fact, whereas genetic ablation of GPR3 reduced Aβ levels, the overexpression of the latter increased Aβ production in Alzheimer’s mouse model^26^. The fact that GPR3 could be the key to find new treatments for the Alzheimer’s Disease, makes this receptor an ideal case of study especially from a structural point of view. Unfortunately, the GPR3 receptor does have neither a known 3D structure nor a known endogenous agonist. Only a study on its constitutive activity has been reported^40^ for this receptor as well as some data on two non-endogenous ligands: (i) an agonist, DPI^34^ and (ii) an inverse agonist, cannabidiol^41^. Although the latter has been recently associated to GPR3, it is not specific for this receptor as it interacts also with a close homolog, GPR6. Thus, here the model of the GPR3 receptor was built based on the active structure of human A_2a_ adenosine receptor (PDB code: 5G53)^42^, using the GOMoDo webserver^43^. The GPR3 receptor shared ~23% of sequence identity with the template and this value was within the range of the identities between the target and its best templates. However, this template turned out to be the most reasonable in terms of MODELLER scores^44^ and its conformational active state^45^, which is expected to be suitable for the agonist-bound state studies (see Methods). The target-template alignment was then manually checked in order to verify the presence of ALL the conserved features of the GPCRs family as the X.50 in each transmembrane helix, the DRY motif in transmembrane helix 3 and the NPxxY motif in transmembrane helix 7^46^. All the conserved features were preserved (SI1) except for the disulfide bridge between the extracellular loop 2 (ECL2) and transmembrane helix 3 (TM3). Indeed, GPR3 has no cysteines in the TM3 (see Methods and SI1 for alignment details).

**Figure 1:**
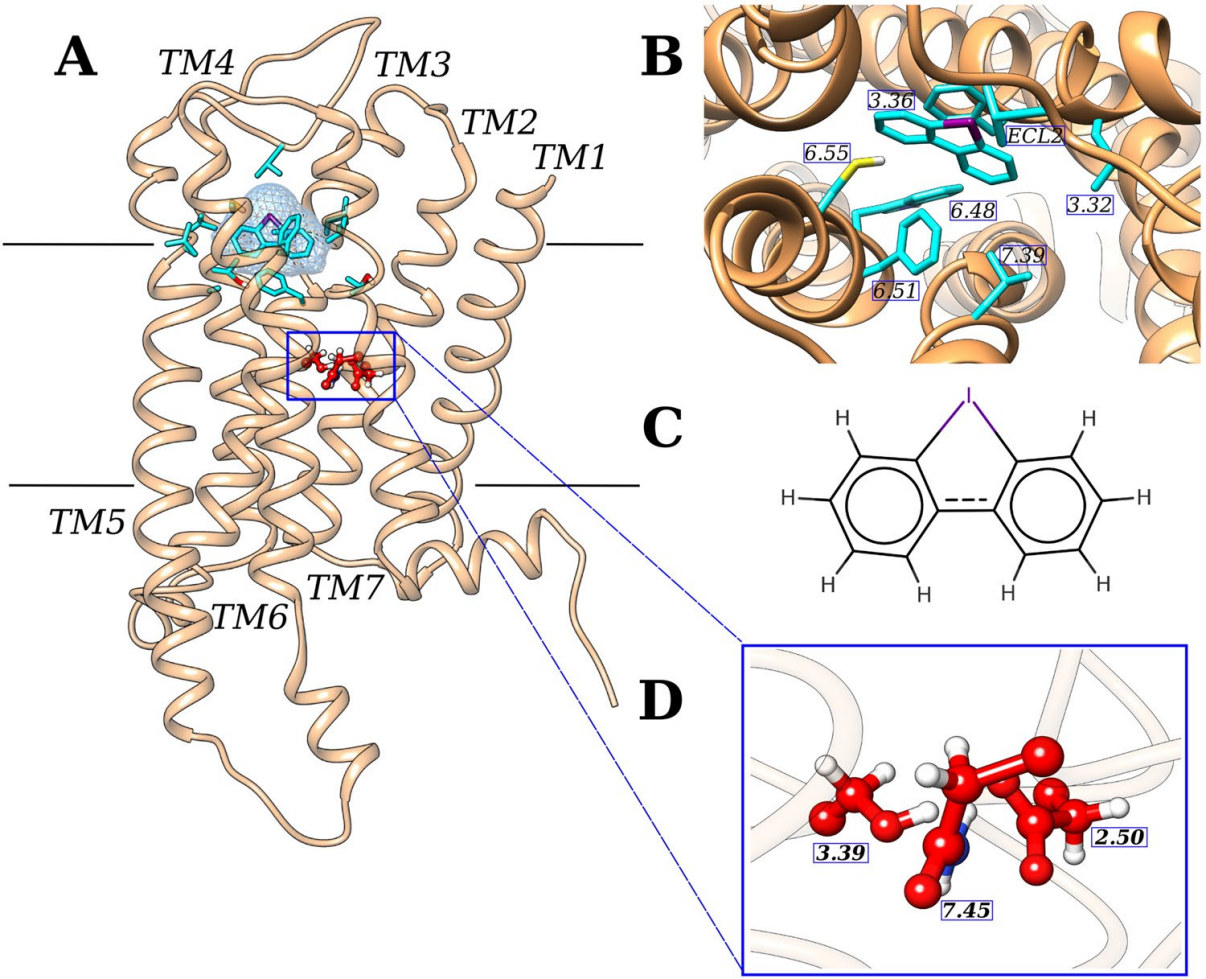
GPR3-DPI complex docking results. In all the panels the GPR3 receptor is shown in salmon and its agonist DPI is shown in cyan. Residues side chains located in the orthosteric binding cavity are shown in cyan, while residues located in the sodium allosteric binding cavity are shown in red. The receptor is oriented with the N-terminus in the extracellular part and the C-terminus in the intracellular part (**A**), DPI and side chains of residues 5 Å distant from the agonist are shown in cyan (**B**), chemical structure of DPI with the iodine atom indicated in violet (**C**), side chains of residues involved in allosteric sodium binding are shown in red (**D**).

The generated model was then used to perform *in silico* docking experiments using the Haddock program^47^ through the GOMoDo webserver. The residues located in the top half part of the receptor (SI2) were predicted as located in the putative binding cavity and used as ambiguous interaction restraints (AIR) for the docking step (see Methods). Once the last docking step was completed, all the complexes (200 in total) were clustered. The best docking GPR3-DPI pose (Fig. 1) was chosen as the one with the lowest HADDOCK score within the most populated cluster. In that conformation, the synthetic agonist DPI is positioned inside the canonical GPCRs orthostatic cavity^48^ (Fig. 1A). GPR3’s putative binding cavity results mostly hydrophobic, with the phenyl rings of DPI interacting with Leu283^7.39^, Leu113^3.32^, Trp260^6.48^, and Val186^ECL2^ (Fig. 1B). Among all the interactions, two specific interactions captured our attention, (i) a halogen-bond interaction^49^ between the iodine atom of DPI and Cys267^6.55^ (~4 Å) and (ii) a ‘sandwich-like’ conformation in which DPI is inserted between two phenylalanine residues, Phe120^3.36^ and Phe263^6.51^ (Fig. 1C).

Nonetheless, due to the low sequence identity and with the aim of better sample the conformational space of the ligand within the putative binding cavity^50–53^, the best complex was funneled to perform molecular dynamics (MD) simulations using a hybrid molecular mechanics/coarse-grained (MM/CG) approach in order to exhaustively explore the conformational space of the ligand, the binding cavity, and the hydration shell (SI3) as in^54,55^. A detailed description of the MM/CG can be found in the SI section: MM/CG technique description.

**Figure 2:**
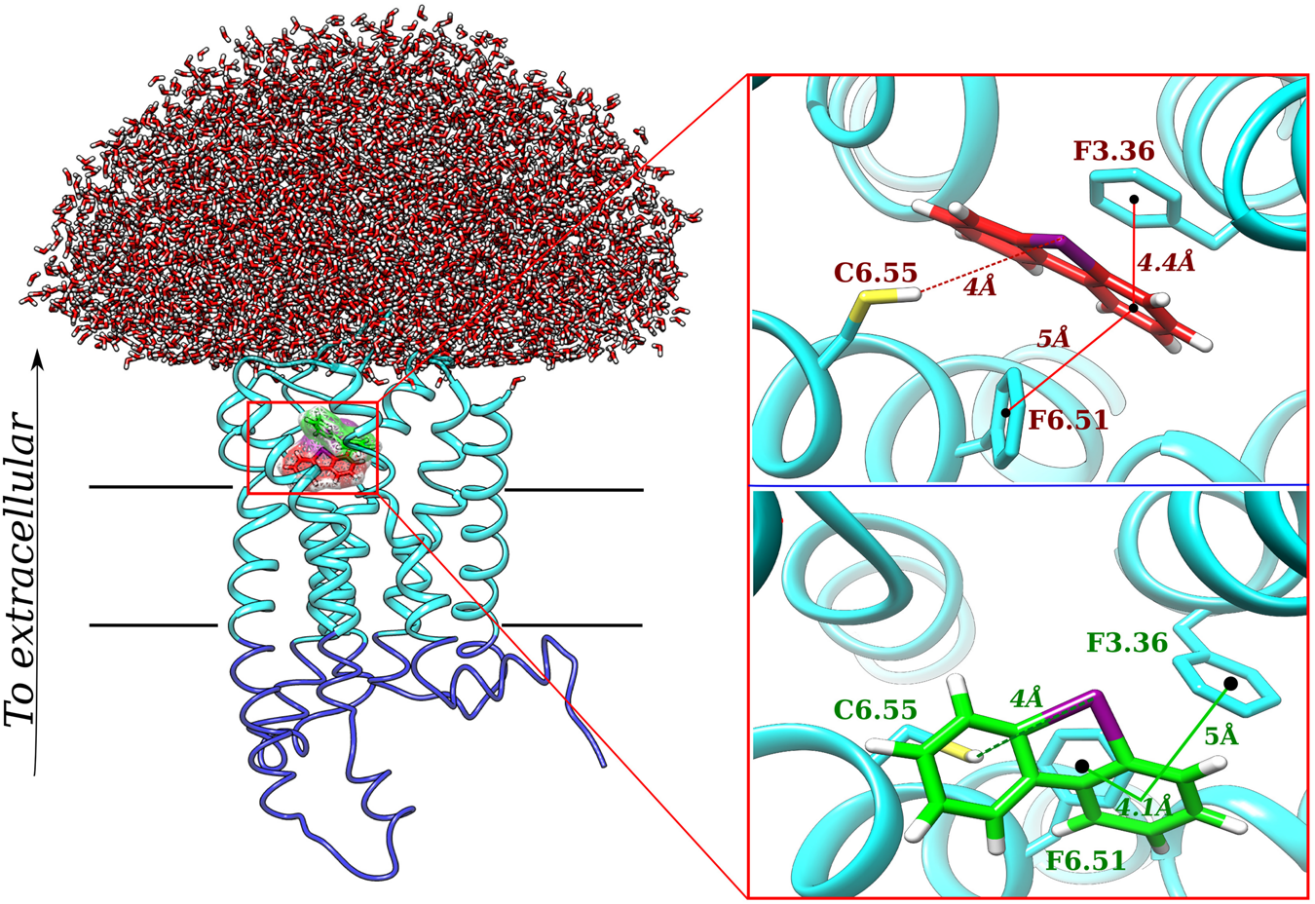
Molecular dynamics results of DPI located in the GPR3 orthosteric binding cavity. DPI-GPR3 complex, together with the water dome that surrounds the The MM and CG regions of the receptors are shown in cyan and blue colors, respectively. The receptor is oriented with the N-terminus in the extracellular part and the C-terminus in the intracellular part. In the top right part the best docking complex is shown. While DPI located in the orthosteric binding cavity of the GPR3 receptor is shown in red,  residues Cys^6.55^, F^3.36^ and F^6.51^  are shown in cyan. In the bottom right, MM/CG simulations of the best complex is represented. DPI is shown in green, while Cys^6.55^, F^3.36^ and F^6.51^ residues are shown in cyan.

The system underwent 700 nanoseconds (ns) of simulations at room temperature, reaching the stability after 300 ns (SI4 and SI5). We then clustered all the trajectory and analyzed the representative conformation of the most populated cluster (Fig. 2A). We noticed very few differences comparing the docking and the simulations results. The simulations relaxed and did not alter the receptor/DPI interactions compared with the initial conformation (Fig. 2B, red color and green color). Indeed, during the simulations, DPI slightly shifted and tilted from its initial position, assuming a non-planar conformation, maintaining however the interaction with Cys267^6.55^ which side chain moved towards to the iodine atom at distance <4 Å. Simulations thus confirmed the *halogen-bond* interaction predicted by docking experiments. Moreover, also other two residues involved in the docking predictions, Phe120^3.36^ and Phe263^6.51^ confirmed their contribution in the ligand binding, shifting the side chains accordingly the DPI rings position (Fig. 2B, green color) and maintaining the π-stacking interactions with the ligand.

Furthermore, we decided to study also the putative sodium (Na^+^) allosteric binding site, that has a fundamental importance in allosteric modulation of GPCRs^50,51^. The residues that mostly contribute to sodium binding along the GPCRs family, i.e S^3.39^, N^7.45^ and D^2.50^ are described in literature as highly conserved. In particular, we observed that residue in position 2.50 (Fig. 1D) is an aspartic acid in 90% of the eukaryotic GPCRs accordingly to the curated multiple sequence alignment of the GPCRdb. This residue can highly modulate the function of GPCRs. The role of sodium modulation is well known for GPCRs^51^. Mutagenesis studies on residues involved in Na^+^ coordination, and in particular Asp^2.50^, highlighted the different effects that allosteric sodium may have in various class A GPCRs signaling^50^. Indeed, Asp^2.50^ replacement with uncharged amino acids can drastically reduce the agonist-induced G protein activation^56–61^ or modulate the allosteric effect of the G-protein on ligand binding^62^. The presence of sodium ions in the allosteric cavity can also exert different effects on the constitutive signaling of GPCRs. In many cases, the presence of bound Na^+^ seems to stabilize the inactive conformation of the receptor reducing the constitutive G-protein^58–60^, whereas in other receptors the substitution of Na^+^ coordinating Asp^250^ abolishes the constitutive G-protein coupling and activation without affecting the agonist-stimulated activity^63^. Exhaustive studies have also revealed that the Na^+^ pocket collapses due to the activation-related movements of the transmembrane helixes^64,65^. In the allosteric binding site, Na^+^ is coordinated by a salt bridge formed with Asp^2.50^ together with other additional polar interactions with Ser^3.39^ and Asn^7.49^. Most of the studies agree with the fact that the constitutive activity can be dramatically affected by mutations in Asp^2.50^ ^66–68^.

In the light of these results together with the results obtained with our simulations, we decided to proceed with *in vitro* experiments to validate/reject our hypothesis.

### Effect of selected single point mutations on GPR3 signaling

**Figure 3:**
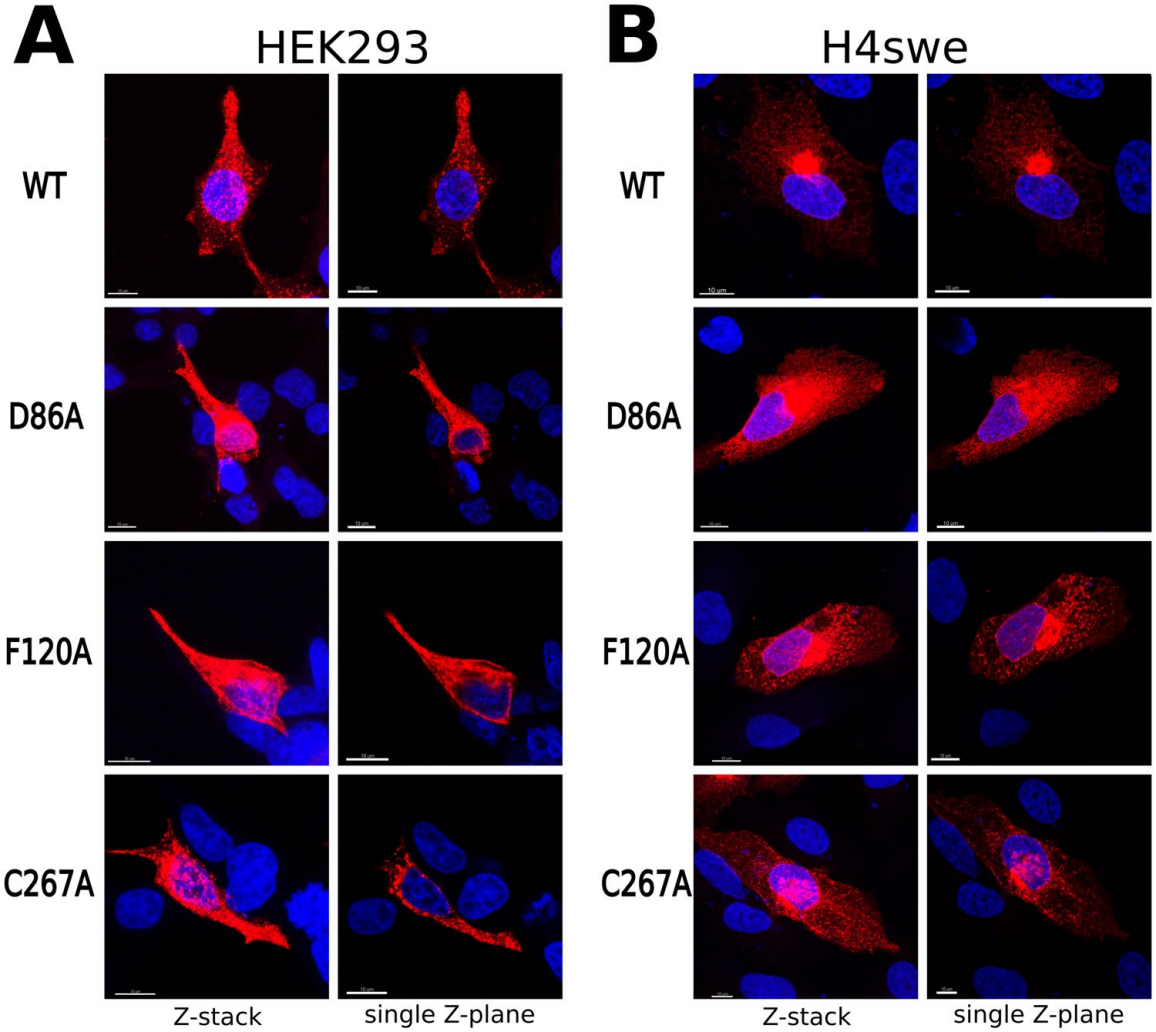
Expression and subcellular localization of WT GPR3 and mutants. Representative confocal images of HEK 293 (**A**) and H4swe (**B**) cells expressing wild-type (WT) or single point mutants of GPR3. Cells were fixed with 4% paraformaldehyde 24 hours post transfection and stained with a monoclonal antibody against the Myc epitope fused to the C-terminal end of the receptor. The nuclei were stained with DAPI (blue). Single Z-planes (right panels) and maximum intensity projections (Z-stack, left panels) are shown for each sample.

We performed wet-lab alanine scanning mutagenesis on residues Cys267^6.55^ and Phe120^3.36^ (SI6), putatively involved in halogen and π-stacking interactions with the ligand, respectively, and on D86^2.50^, the highly conserved acidic residue present in the putative allosteric Na^+^ binding site. The three mutants were first tested for expression and localization in eukaryotic cells. Figure 3 shows representative immunofluorescence confocal images of HEK293 (A) and H4swe (B) cells expressing GPR3 wild type and D86A, F120A and C267A mutants. In all the cases, the receptor is clearly distinguishable in the cells, with no appreciable difference in fluorescence intensity and distribution between the WT protein and the three mutants. These results indicate that the mutations of these residues do not hamper the expression nor the correct folding and trafficking of the receptor in the cells.

**Figure 4:**
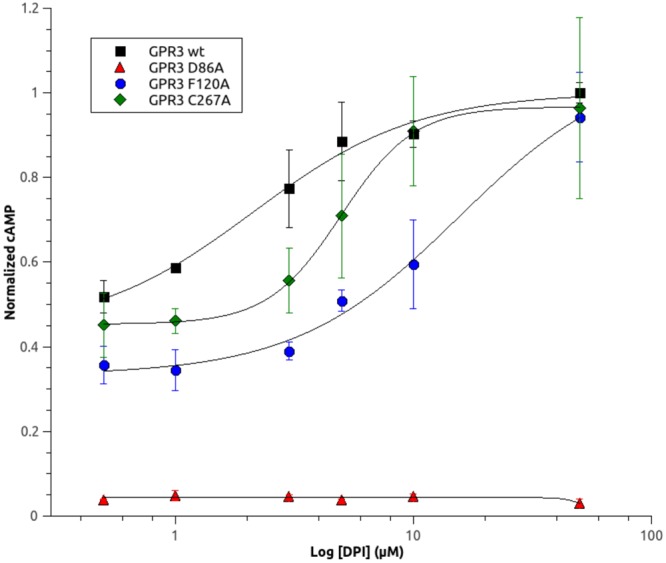
Effect of single point mutations on DPI-induced activation of GPR3. Dose-response curves for DPI in HEK293 cells expressing WT and single point mutants of GPR3. Twenty four hours after transfection, cells were stimulated for 30 minutes with increasing concentration of DPI and the intracellular cAMP level was measured. cAMP values were normalized to the maximal response. Nonlinear regression analysis was performed to generate dose-response curves and calculate concentration for 50% of the maximal effect (EC_50_). Data are the mean ± SD of 3 independent experiments.

Thus, we investigated the effect of the three mutations on the constitutive and DPI-induced activation of GPR3. Figure 4 displays dose-response curves obtained measuring the cAMP concentration in HEK293 cells transfected with WT or mutant receptors and treated with increasing concentration of DPI. The deletion of Cys267^6.55^ or Phe120^3.36^ side chains, predicted by *in silico* experiments to be involved in DPI binding, has the effect to increase the DPI EC_50_ from ~2 µM in GPR3 WT to 5 µM and 15 µM, respectively. This can be explained by a decreased affinity for the agonist due to a reduction of molecular contacts in the binding cavity when mutants are introduced, and further supports the accuracy of our model. Indeed, we performed also an i*n silico* docking between the mutated receptor and the DPI and we noticed a reduction in the number of complexes in the most populated cluster (SI7), hampering the choice of one single structure as the representative of the most populated cluster. Indeed, the number of clusters increased and the number of structures within the clusters reduced significantly, thus no clear solution is offered.

Conversely to the previous two mutants, the mutation in alanine of Asp86^2.50^, putatively involved in the allosteric Na^+^ binding site completely abolished either the constitutive and DPI-induced stimulation of adenylyl cyclase by GPR3, suggesting that this mutation produces a totally inactive form of the receptor. These results point out that binding of allosteric Na^+^ is essential for GPR3 to maintain its constitutive activity or to assume an active conformation.

**Figure 5:**
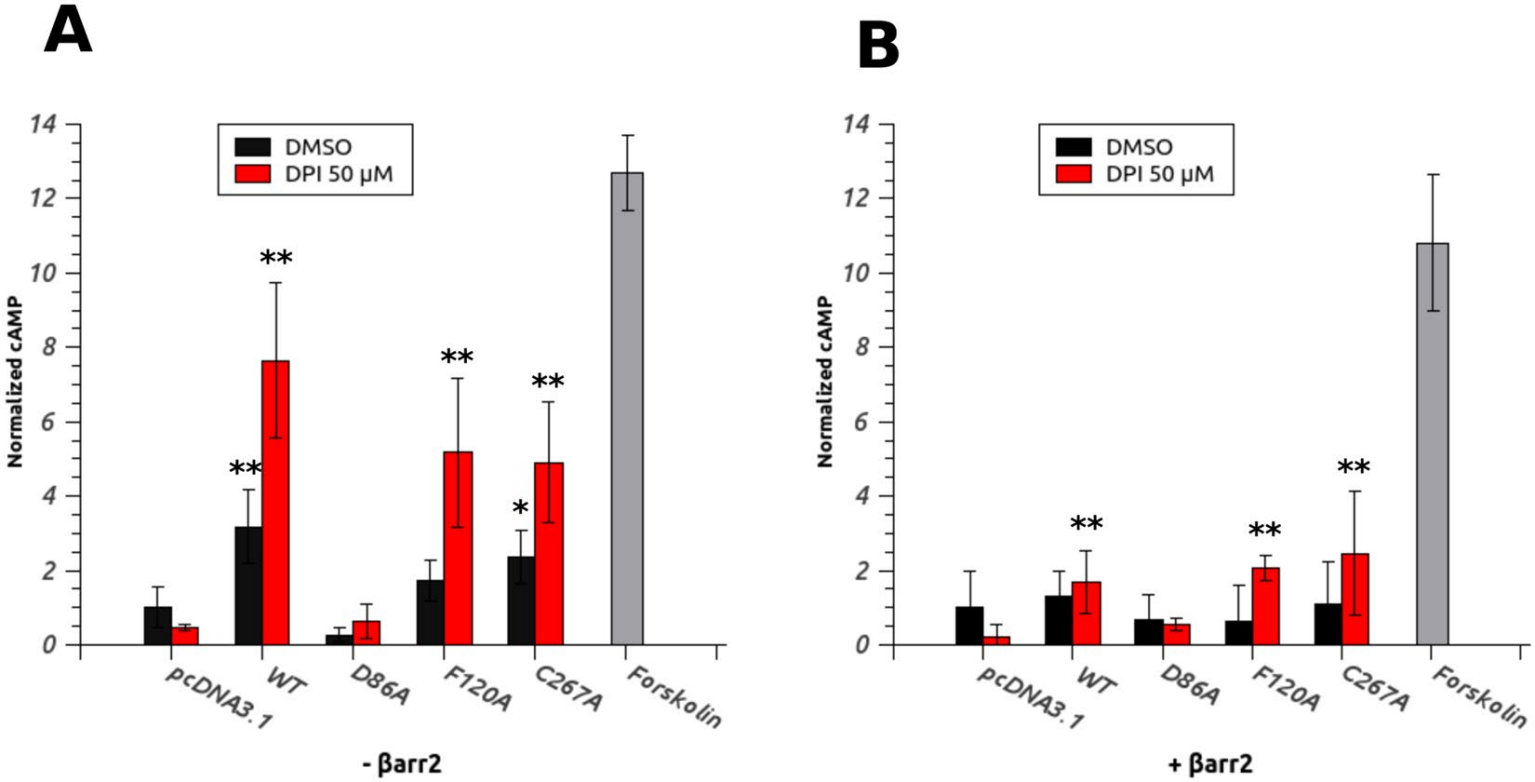
βarr2 decreases constitutive and DPI-induced activity of GPR3. (**A**) HEK293 cells transfected with empty vector (pcDNA3.1), WT GPR3 and single point mutants were treated with DMSO or 50 μM DPI for 30 minutes and the intracellular cAMP level was measured. (**B**) The same experiment as in A was performed in presence of co-transfected human βarr2. 10 μM Forskolin, a known adenylate cyclase activator, was used as a positive control. Data are the mean ± SD of four independent experiments. cAMP values were normalized to the control (empty vector untreated). Statistical significance was determined by one-way ANOVA with Bonferroni-Holmes post-hoc test comparing all samples with the relative control (empty vector) (*p < 0.05, **p < 0.01).

Next, we investigated the effect of constitutive and agonist-induced activity of our mutants on βarr2 interaction. β-arrestin proteins are ubiquitous modulators of GPCRs signaling that recognize and bind to specific phosphorylated residues in the C-terminal tail of active GPCRs and antagonize the interaction with the G-protein. This promotes the desensitization and the internalization of the receptor. HEK293 cells transfected with the WT receptor or three mutants were treated 30 minutes with DMSO (vehicle control) or 50 μM DPI and the amount of intracellular cAMP was determined. Compared with the empty vector control, the constitutive activity of the WT receptor results in a 3-fold increase in cAMP level in unstimulated cells, whereas the expression of F120A and C267A mutants produces a lower, but still significant, increase of cAMP (Fig. 5A, black bars). Upon DPI stimulation, an up to 10-fold increment in cAMP concentration is observed for the WT receptor and 5 to 6-fold for F120A and C267A (Fig. 5A, red bars). Again, neither constitutive nor DPI-induced activity is detected for D86A mutant. When the same experiment is conducted in presence of co-expressed βarr2 (Fig. 5B), a sensible decrease in constitutive activity of WT, F120A and C267A mutants is observed, while no considerable effect can be detected for control and D86A mutated receptor. DPI stimulation still produces an increase in the cAMP level compared to the control (except for D86A mutant), but remarkably lower than in absence of βarr2. These findings suggest that, like WT GPR3, activated F120A and C267A mutants are negatively modulated by βarr2-mediated desensitization, whereas D86A, being totally inactive and therefore likely not-phosphorylated by G protein-coupled receptor kinases (GRKs), is probably unable to interact with βarr2 and this does not allow to appreciate any modulating effect. D68A mutant, although retaining proper folding and localization, appears to be completely unable to stimulate adenylate cyclase both in constitutive conditions and upon agonist stimulation. In this view, the presence of the highly conserved negatively charged Asp^250^, and consequently a complete and functional allosteric Na^+^ binding pocket, seems to be essential for this receptor to maintain its constitutive activity and to activate Gs-protein for downstream signaling. The unraveling of the functional role of the sodium ion in the activation of GPR3 certainly deserves a deeper investigation.

### GPR3-stimulated Aβ production is correlated with receptor activation

**Figure 6:**
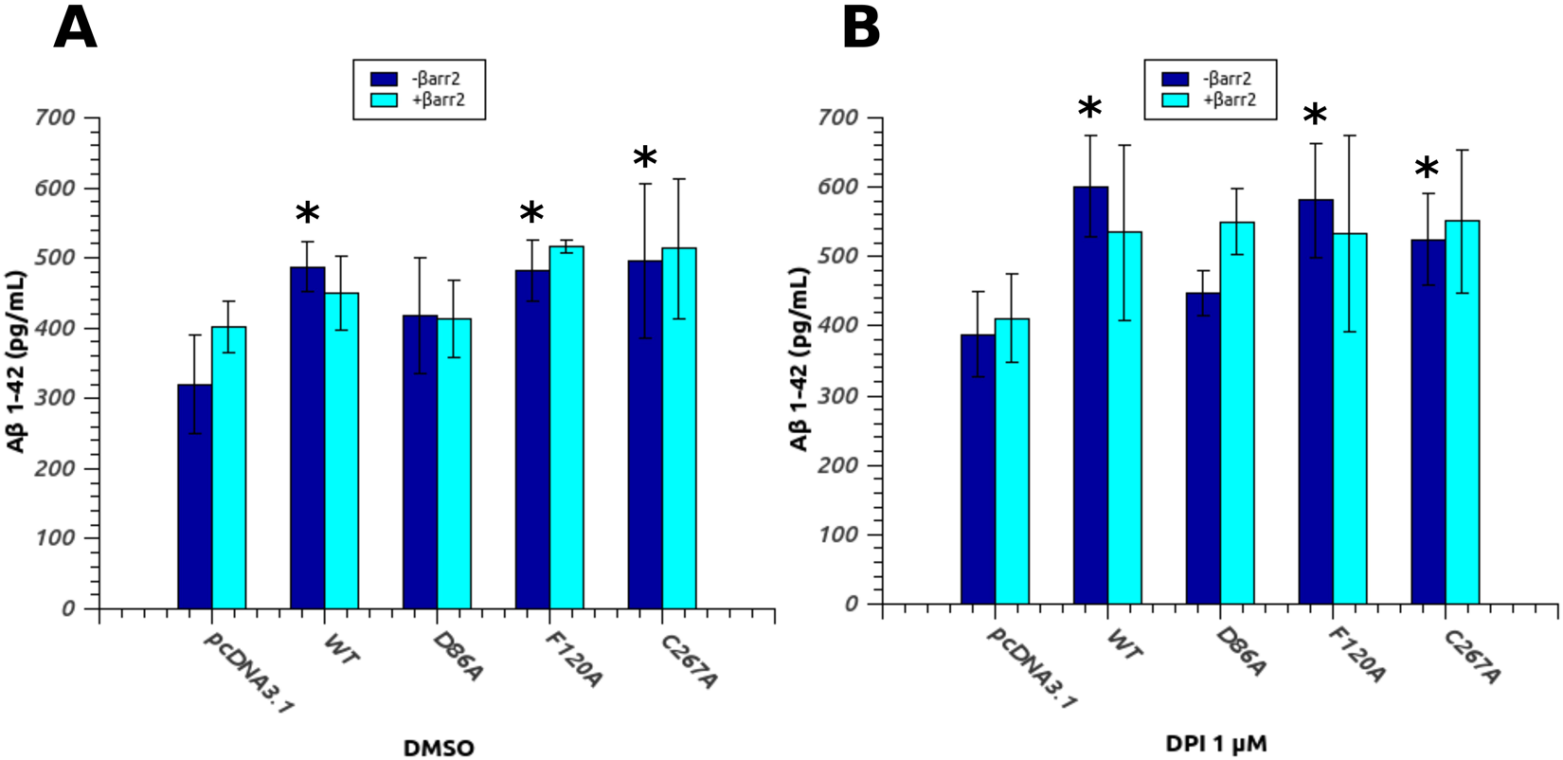
D86A mutation reduces the GPR3-stimulated Aβ production in H4swe cells. H4swe cells transfected with empty vector (pcDNA3.1), WT GPR3 and single point mutants, either in absence (blue bars) or in presence (cyan bars) of co-transfected human βarr2, were treated with DMSO (**A**) or 1 μM DPI (**B**) for 24 hours and the amount of Aβ 1–42 in the culture medium was determined by ELISA. Data are the mean ± SD of three independent experiments. Statistical significance was determined by one-way ANOVA with Bonferroni-Holmes post-hoc test comparing all samples with the relative control (empty vector) (*p < 0.05).

One of the most interesting features of GPR3 is its implication in the APP processing and Aβ amyloid secretion. The first evidence of its role in the amyloid beta production process has been reported for the first time in 2009^26^, where the authors highlighted its ability to up-regulate the γ-secretase activity and Aβ accumulation in neuronal cultures and in animal models. Subsequent works demonstrated that GPR3 modulation of APP cleavage is independent by G-protein coupling but is rather correlated to the recruitment and interaction of the receptor with βarr2^69^. Therefore, we investigated the correlation between the modulating effects of our mutants on GPR3 activity and the stimulated production of Aβ peptides. H4swe cells, expressing the Swedish mutation (K595N/M596L) of amyloid precursor protein (APP-swe), were transfected with WT or mutated GPR3 (alone or in presence of co-transfected βarr2) and the amount of Aβ 1–42 released in the culture medium was measured by ELISA 24 hours after transfection (Fig. 6A). As expected, a statistically relevant (p < 0.05) increment of Aβ with respect to the control is observed for WT GPR3, F120A and C267A, while the inactive mutant D86A does not significantly increase the amount of secreted amyloid peptide. When βarr2 is co-expressed with the receptor (cyan bars), the picture is less clear. βarr2 induces a slight increment in the Aβ 1–42 level in the control (probably modulating other signaling pathways in the cells) but does not affect significantly the amount of peptide produced in cells transfected with WT receptor or mutants. As a result, we could not detect any statistically relevant effect of βarr2 on GPR3 in this cellular model. To further assess the correlation between the activation of GPR3 and its ability to enhance the production of Aβ, we performed the same experiment in presence of an agonist. Due to the lack of knowledge regarding its physiological ligand we used DPI, the only known compound able to activate GPR3. Due to DPI poor selectivity (e.g. it is known to strongly inhibit nitric oxide synthetase from macrophages and endothelial cells and other flavoenzymes) and cell toxicity, even at micromolar concentration, DPI does not have any potential therapeutic application, but it is a useful experimental tool in studying GPR3 signaling *in vitro*. In this case, the transfected cells were incubated 24 hours with 1 μM DPI (a prolonged exposure to higher concentration of DPI resulted in higher cell toxicity, Figure SI8) and the amount of Aβ 1–42 was quantified as before (Fig. 6B). Compared to the empty vector control, DPI stimulation produces a ~50% increase in the amyloid peptide level for WT and, to a less extent, for F120A and C267A, whilst had no influence on D86A. Again, co-transfection with βarr2 produces no appreciable difference in the production of Aβ promoted by WT GPR3 or mutants and the control in these cell line. Taken together, our results prompt us to suggest that there is a correlation between the permanence of the receptor in the active state and its modulation role on γ-secretase complex, although this process has been reported to be independent of G-protein activation. Indeed, DPI stimulation proportionally increases Aβ production in WT and agonist-sensitive mutants, while D86A mutant, devoid of any cAMP stimulation activity and unable to gain access to the active state, is also ineffective in stimulating the production of amyloid peptides. Once activated, GPCRs are phosphorylated at specific positions by GPR kinases that specifically recognize the active form of the receptor and this modification considerably increase the recruitment and binding of β-arrestins. In this view, although indirectly, our findings further support the hypothesis of the involvement of β-arrestin mediated desensitization/internalization pathway in GPR3 modulation of Aβ secretion.

## Conclusions

In this work, we built the first homology model of GPR3 and performed combined docking/molecular dynamics simulations to investigate structural features of this receptor and its binding site. The predictive power of our model allowed the characterization of the GPR3 binding cavity with detailed analysis of GPR3 interaction with DPI and of the Na^+^ binding site, unraveling unexpected effects of the Asp^2.50^ mutation on GPR3 activity and Aβ production. These results brought the light on new possible targets for AD, paving the way for prevention and new therapeutic interventions in Alzheimer’s disease.

## Methods

### Homology modeling

The crystal structure of the GPR3 receptor is not available yet, thus homology modeling technique was carried out to predict its 3D structure. The sequence was retrieved from the Uniprot database (Uniprot entry: P46089) and the model was generated through the GOMoDo web-server, as in^52,53,70,71^. First, a multiple sequence alignment was generated by the GOMoDo webserver to create a Hidden Markov Model (HMM) for our target. The resulting HMM of the GPR3 target were aligned against all the HMMs of the GPCR templates available in the GOMoDo webserver, using HHsearch 2.0.16 algorithm^66^. The use of profile-profile HMMs alignments is known to improve the target-template alignment especially if the latter has a low sequence identity^69^, ~23%, as in our case. Then 100 models were generated for each target-template pair using MODELLER 9v10^72^. MODELLER quality scores were used to evaluate the receptor models based on DOPE (lower better) and GA341 (higher better) values^73,74^. Among all the templates, the human A_2A_ adenosine receptor (PDB code: 5G53, resolution: 2.79 Å) was identified as the most suitable one (see also Results section). On one hand, the models generated with A_2A_ adenosine receptor as template showed better MODELLER quality scores^73,74^ for all the hChem-GPCRs object of this study. On the other, this template was solved in a fully active state^45,46^, which is expected to be the agonist-bound conformational state. The template-target alignment was then checked by hand, in order to preserve the conserved features of class A GPCRs (SI1). We use the GPCRdb generic number position^36^ to have a coherent numeration of the residues between the target and the template. The chosen model was further considered for docking.

### Docking Experiments

The agonist DPI, was docked on the final GPR3 receptor model using the HADDOCK v2.1^47^ pipeline through the GOMoDO webserver^43^. Since the GPR3 has never been characterized at a binding level, first the putative binding cavity residues (SI2) were predicted using the Fpocket server^75^. Then, these residues, that covered the top half part of the protein, were used as active residues to guide the docking experiments. The structure of DPI was manually designed using the MarviSketch tool (http://www.chemaxon.com) and was initially parametrized using the PRODRG server^76^. After we noticed that the partial charges were wrongly attributed, we parametrize again the ligand with ACPYPE (ACPYPE - AnteChamber PYthon Parser interface) and manually changed the partial charges in the PRODRG files (see SI9 and SI10). 1000 random structures were generated through the first initial rigid docking step. Then, after an initial ranking, the best 200 complexes underwent through the refinement step with both ligand and receptor treated as flexible. The resulting receptor-agonist complexes were clustered using an RMSD cutoff of 1.0 Å and the complex of the most populated cluster with the lowest HADDOCK score underwent 700 ns of molecular mechanics/coarse-grained MM/CG molecular dynamics simulations^54,55^.

### Molecular Dynamics Simulations

The complex was divided into two parts i) a MM part, which includes the extracellular region of GPR3, agonist and the residues in the binding cavity and ii) a CG part, containing the lower half part of the protein^55^. Since GPR3 is a membrane receptor the presence of the lipid bilayer in our case was taken into account introducing a wall located at 2.0 Å from the protein’s C*α* atoms. The MM region was described with the GROMOS 96 force field, water was described with the SPC force field and the CG part was described using a Go-like potential as in^52–55^. In order to keep fixed the distance of bonds containing hydrogen(s) we used the SHAKE algorithm. The simulations were performed at a constant temperature (300K). Once completed the simulation, a clustering analysis was performed. For this purpose, all the protein backbone was aligned, and all the conformations were clustered according to the position of the ligand. A 1 Å cut-off was used to group different frames in the same cluster as in^52–55^.

### DNA Constructs

The complete GPR3 sequence was isolated from a human blood sample containing leucocytic DNA. The sequence, amplified by PCR, was TA-cloned into the pGEM-T Easy Vector and sequence-verified. For mammalian expression, GPR3 was subcloned into the pcDNA 3.1/myc-His A vector in frame with a C-terminal myc epitope followed by a 6XHis tag (GPR3-pcDNA). All GPR3 mutants were created with a QuikChange Site-Directed Mutagenesis Kit (Stratagene) and verified by DNA sequencing. The sequence of full length human b-arrestin2 was obtained from a HEK293 cDNA library. Briefly, 1 ug of total RNA was used to synthesize the first cDNA strand by means of the Superscript First Strand Synthesis System (Invitrogen) using an Oligo(dT) 18 primer. b-arrestin2 (barr2) sequence was amplified by PCR using a High Fidelity Taq DNA polymerase (Jena Biosciences) and subcloned into the pEGFP-N1 vector in frame with a C-terminal EGFP tag (barr2-pEGFP). The construct was verified by DNA sequencing.

### Cell Culture and Transfection

Cell lines were grown in monolayer in Dulbecco’s modified Eagle’s medium (DEMEM) supplemented with 10% (v/v) FBS, 100 U/ml penicillin, 100 μg/ml streptomycin, 2 mM glutamine and 1% (v/v) NEAA (HEK293) or OptiMEM medium supplemented with 10% (v/v) FBS, 200 U/ml penicillin and 200 μg/ml streptomycin (H4swe) at 37 °C in a moist incubator supplied with 5% CO_2_ atmosphere. For transfection, cells were seeded at a density of 15000 cell/well (for 96 well plates) or 45000 cells/well (for 24 well plates) in OptiMEM medium supplemented with 10% (v/v) FBS and let to attach overnight. The next day, cells were transfected with the appropriate vectors (50 ng of DNA/well for 96 well plates or 250 ng of DNA/well for 24 well plates) using Lipofectamine LTX (ThermoFisher) reagent according to the to manufacturer’s instructions.

### Immunostaining and Confocal microscopy analysis

For confocal microscopy, 5 × 10 5 cells/well were seeded onto 18 mm round coverslips in a 24-well plate and left to attach overnight. The next day, the medium was changed to Optimem supplemented with 10% (v/v) FBS and the cells were transfected with 250 ng of DNA as described in the previous section. Twenty four hours after transfection, the cells were washed twice with PBS and fixed with 4% paraformaldehyde for 20 min. Fixed cells were treated for 10 min with blocking solution (2% BSA, 2% normal goat serum (NGS), 0.2% Triton X100 in PBS) and incubated overnight with anti-myc primary antibody (Roche) diluted 1:100 in blocking solution. After three washes with PBS, samples were incubated with AlexaFluor-568 goat anti-mouse secondary antibody (ThermoFisher) diluted 1:2000 for 1 hour. After final washes, coverslips were treated with a 4′6-diamidino- 2-phenylindole (DAPI) solution (100 ng/mL) for 10 min at room temperature. The slips were fixed onto glass slides with a drop of anti-fading mounting medium and sealed with nail polish. Images at different Z-planes were collected on a Leica tcs-sp5 confocal microscope. 405 and 543 nm lasers were used for the excitation of DAPI and Alexafluor dye respectively. Images were processed with the software Imaris (Bitplane AG) or ImageJ (ref.^1^).

### cAMP accumulation assay

HEK 293 cells were seeded at a density of 15,000 cells per well in a 96 well plate and left to attach overnight. The next day, cells were transfected with 50 ng of plasmidic DNA (pcDNA3.1, WT GPR3-pcDNA and mutants, either with or without barr2-pEGFP) as described above. Forty eight hours after transfection, cells were incubated with 100 μl of HHBS containing 1 mM IBMX (phosphodiesterase inhibitor) at 37 °C and for 30 minutes and stimulated with 1 μL of DPI (or forskolin) for additional 30 minutes. DPI and forskolin were dissolved in DMSO at different concentrations, according to the experimental design. The medium was removed and 50 μl of ice cold 100% ethanol was added to each well. After evaporation, cells were resuspended in 50 μl of Lysis buffer (0.3% Tween 20 in HHBS buffer) and incubated at room temperature for 10 minutes. The cAMP concentration in the cell lysate (5 μL) was determined with a cAMP AlphaScreen Assay kit (Perkin Elmer) and a EnSpire Alpha Plate Reader (Perkin Elmer) according to the manufacturer’s instructions.

### Aβ measurements in H4swe culture medium

H4swe cells were seeded at a density of 15,000 cells per well in a 96 well plate and left to attach overnight. The next day, cells were transfected with 50 ng of plasmidic DNA (pcDNA3.1, WT GPR3-pcDNA and mutants, either with or without barr2-pEGFP) as described above. Twenty four hours after transfection, the medium was replaced with 150 mL of Optimem without serum, either in presence or in absence of 1 mM DPI, and the cells were incubated for additional 24 hours at 37 °C. At the end of the treatment the concentration of Aβ 1–42 in the culture medium was determined with a commercial ELISA kit (Life Technologies).

### Statistical analysis

All values shown are the mean +/− SD of three or four independent experiments. Statistical significance was determined using one-way ANOVA with Bonferroni-Holmes post-hoc test (multiple comparison and comparison of all sets with control).

### Data availability

Data generated or analyzed during the current study are available from the corresponding author upon request.

## Electronic supplementary material

Supplementary Information

## References

[1] Fredriksson R, Lagerström MC, Lundin LG, Schiöth HB (2003). The G-protein-coupled receptors in the human genome form five main families. Phylogenetic analysis, paralogon groups, and fingerprints. Mol Pharmacol.

[2] Lagerström MC, Schiöth HB (2008). Structural diversity of G protein-coupled receptors and significance for drug discovery. Nat Rev Drug Discov.

[3] Lefkowitz RJ (2004). Historical review: a brief history and personal retrospective of seven-transmembrane receptors. Trends Pharmacol Sci.

[4] Spiegel AM (2004). Inherited diseases involving G proteins and G protein-coupled receptors. Annu Rev Med.

[5] Tan CM (2004). Membrane trafficking of G protein-coupled receptors. Annu Rev Pharmacol Toxicol.

[6] Rask-Andersen M, Almén MS, Schiöth HB (2011). Trends in the exploitation of novel drug targets. Nature reviews Drug discovery.

[7] Wacker D, Stevens RC, Roth BL (2017). How ligands illuminate GPCR molecular pharmacology. Cell.

[8] Hauser AS, Attwood MM, Rask-Andersen M, Schiöth HB, Gloriam D (2017). E.Trends in GPCR drug discovery: new agents, targets and indications. Nature Reviews Drug Discovery.

[9] Borroto-Escuela DO (2017). Understanding the role of GPCR heteroreceptor complexes in modulating the brain networks in health and disease. Frontiers in cellular neuroscience.

[10] Ballard C (2011). Alzheimer’s disease. Lancet.

[11] Querfurth HW, LaFerla FM (2010). Alzheimer’s disease. N Engl J Med.

[12] Association A’s (2016). Alzheimer’s disease facts and figures. Alzheimers Dement.

[13] Qiu C, Kivipelto M, Von Strauss E (2009). Epidemiology of Alzheimer’s disease: occurrence, determinants, and strategies toward intervention. Dialogues Clin Neurosci.

[14] Niu H (2017). Trends of mortality from Alzheimer’s disease in the European Union, 1994–2013. Eur J Neurol.

[15] Carlesimo GA, Oscar-Berman M (1992). Memory deficits in Alzheimer’s patients: a comprehensive review. Neuropsychol Rev.

[16] Landes AM, Sperry SD, Strauss ME, Geldmacher DS (2001). Apathy in Alzheimer’s disease. J Am Geriatr Soc.

[17] Bäckman L, Jones S, Berger AK, Laukka EJ, Small BJ (2004). Multiple cognitive deficits during the transition to Alzheimer’s disease. J Intern Med.

[18] Förstl H, Kurz A (1999). Clinical features of Alzheimer’s disease. Eur Arch Psychiatry Clin Neurosci.

[19] Pensalfini A (2014). Intracellular amyloid and the neuronal origin of Alzheimer neuritic plaques. Neurobiology of disease.

[20] Selkoe DJ (2002). Alzheimer’s disease is a synaptic failure. Science.

[21] Annaert W, De Strooper B (2002). A cell biological perspective on Alzheimer’s disease. Annu Rev Cell Dev Biol.

[22] Haass C (2004). Take five–BACE and the gamma-secretase quartet conduct Alzheimer’s amyloid beta-peptide generation. EMBO J.

[23] Wilquet V, De Strooper B (2004). Amyloid-beta precursor protein processing in neurodegeneration. Curr Opin Neurobiol.

[24] Haass C (1995). The Swedish mutation causes early-onset Alzheimer’s disease by beta-secretase cleavage within the secretory pathway. Nat Med.

[25] Jonsson A (2012). mutation in APP protects against Alzheimer’s disease and age-related cognitive decline. Nature.

[26] Thathiah A (2009). The orphan G protein-coupled receptor 3 modulates amyloid-beta peptide generation in neurons. Science.

[27] Iismaa TP (1994). Isolation and chromosomal localization of a novel human G-protein-coupled receptor (GPR3) expressed predominantly in the central nervous system. Genomics.

[28] Miyagi T, Tanaka S, Hide I, Shirafuji T, Sakai N (2016). The Subcellular Dynamics of the Gs-Linked Receptor GPR3 Contribute to the Local Activation of PKA in Cerebellar Granular Neurons. PLoS One.

[29] Mehlmann LM (2004). The Gs-linked receptor GPR3 maintains meiotic arrest in mammalian oocytes. Science.

[30] Hinckley M, Vaccari S, Horner K, Chen R, Conti M (2005). The G-protein-coupled receptors GPR3 and GPR12 are involved in cAMP signaling and maintenance of meiotic arrest in rodent oocytes. Dev Biol.

[31] Eggerickx D (1995). Molecular cloning of an orphan G-protein-coupled receptor that constitutively activates adenylate cyclase. Biochem J.

[32] Uhlenbrock K, Gassenhuber H, Kostenis E (2002). Sphingosine 1-phosphate is a ligand of the humangpr3, gpr6 and gpr12 family of constitutively active G protein-coupled receptors. Cell Signal.

[33] Yin H (2009). Lipid G protein-coupled receptor ligand identification using beta-arrestin PathHunter assay. J Biol Chem.

[34] Ye C (2014). Identification of a novel small-molecule agonist for human G protein-coupled receptor 3. J Pharmacol Exp Ther.

[35] Nelson CD, Sheng M (2013). Gpr3 stimulates Aβ production via interactions with APP and β-arrestin2. PLoS One.

[36] Isberg V (2017). GPCRdb: an information system for G protein-coupled receptors. Nucleic Acids Res.

[37] Tourino C (2012). The orphan receptor GPR3 modulates the early phases of cocaine reinforcement. Br J Pharmacol.

[38] Valverde O (2009). GPR3receptor, a novel actor in the emotional-like responses. PloS One.

[39] Wolfe MS (2013). Alzheimer’s γ-secretase under arrestin. Nat Med.

[40] Martin AL, Steurer MA, Aronstam RS (2015). Constitutive Activity among Orphan Class-A G Protein Coupled Receptors. PLoS One.

[41] Laun AS, Song ZH (2017). GPR3 and GPR6, novel molecular targets for cannabidiol. Biochem Biophys Res Commun.

[42] Carpenter. B (2016). Structure of the adenosine A(2A) receptor bound to an engineered G protein. Nature.

[43] Sandal M (2013). GOMoDo: A GPCRs online modeling and docking webserver. PLoS One.

[44] Webb B, Sali A (2016). Comparative Protein Structure Modeling Using MODELLER. Curr Protoc Protein Sci.

[45] Venkatakrishnan AJ (2016). Diverse activation pathways in class A GPCRs converge near the G-protein-coupling region. Nature.

[46] Venkatakrishnan AJ (2013). Molecular signatures of G-protein-coupled receptors. Nature.

[47] Dominguez C, Boelens R, Bonvin AM (2003). HADDOCK: a protein-protein docking approach based on biochemical or biophysical information. J Am Chem Soc.

[48] Suku E, Giorgetti A (2017). Common evolutionary binding mode of rhodopsin-like GPCRS: Insights from structural bioinformatics, AIMS Press, AIMS. Biophysics.

[49] Arman HD, Gieseking RL, Hanks TW, Pennington WT (2010). Complementary halogen and hydrogen bonding: sulfur…iodine interactions and thioamide ribbons. Chem Commun (Camb).

[50] Katritch V (2014). Allosteric sodium in class A GPCR signaling. Trends Biochem Sci.

[51] Liu W (2012). Structural basis for allosteric regulation of GPCRs by sodium ions. Science.

[52] Fierro F (2017). Agonist Binding to Chemosensory Receptors: A Systematic Bioinformatics Analysis. Front Mol Biosci.

[53] Sandal M (2015). Evidence for a Transient Additional Ligand Binding Site in the TAS2R46 Bitter Taste Receptor. J Chem Theory Comput.

[54] Leguèbe M (2012). Hybrid molecular mechanics/coarse-grained simulations for structural prediction of G-protein coupled receptor/ligand complexes. PLoS One.

[55] Neri M (2005). Coarse-grained model of proteins incorporating atomistic detail of the active site. Phys Rev Lett.

[56] Bihoreau C (1993). Mutation of Asp74 of the rat angiotensin II receptor confers changes in antagonist affinities and abolishes G-protein coupling. Proc Natl Acad Sci USA.

[57] Perlman JH (1997). Interactions between conserved residues in transmembrane helices 1, 2, and 7 of the thyrotropin-releasing hormone receptor. J Biol Chem.

[58] Rose PM (1995). Aspartate mutation distinguishes ETA but not ETB receptor subtype-selective ligand binding while abolishing phospholipase C activation in both receptors. FEBS Lett.

[59] Prossnitz ER, Schreiber RE, Bokoch GM, Richard DY (1995). Binding of low affinity N-formyl peptide receptors to G protein. Characterization of a novel inactive receptor intermediate. J Biol Chem.

[60] Tao Q, Abood ME (1998). Mutation of a highly conserved aspartate residue in the second transmembrane domain of the cannabinoid receptors, CB1 and CB2, disrupts G-protein coupling. J Pharmacol Exp Ther.

[61] Wang CD, Gallaher TK, Shih JC (1993). Site-directed mutagenesis of the serotonin 5-hydroxytrypamine2 receptor: identification of amino acids necessary for ligand binding and receptor activation. Mol Pharmacol.

[62] Li B (2005). Random mutagenesis of the M3 muscarinic acetylcholine receptor expressed in yeast: identification of second-site mutations that restore function to a coupling-deficient mutant M3 receptor. J Biol Chem.

[63] Nie J, Lewis DL (2001). Structural domains of the CB1 cannabinoid receptor that contribute to constitutive activity and G-protein sequestration. J Neurosci.

[64] Gutiérrez-de-Terán H (2013). The role of a sodium ion binding site in the allosteric modulation of the A(2A) adenosine G protein-coupled receptor. Structure.

[65] Rasmussen SG (2011). Crystal structure of the β2 adrenergic receptor-Gs protein complex. Nature.

[66] Quitterer U, AbdAlla S, Jarnagin K, Müller-Esterl W (1996). Na + ions binding to the bradykinin B2 receptor suppress agonist-independent receptor activation. Biochemistry.

[67] Seifert R, Wenzel-Seifert K (2001). Unmasking different constitutive activity of four chemoattractant receptors using Na + as universal stabilizer of the inactive (R) state. Receptors Channels.

[68] Seifert R, Wenzel-Seifert K (2002). Constitutive activity of G-protein-coupled receptors: cause of disease and common property of wild-type receptors. Naunyn Schmiedebergs Arch Pharmacol.

[69] Söding J, Biegert A, Lupas AN (2005). The HHpred interactive server for protein homology detection and structure prediction. Nucleic Acids Res.

[70] Radu BM (2017). All muscarinic acetylcholine receptors (M1-M5) are expressed in murine brain microvascular endothelium. Sci Rep.

[71] Marchiori A (2013). Coarse-grained/molecular mechanics of the TAS2R38 bitter taste receptor: experimentally-validated detailed structural prediction of agonist binding. PLoS One.

[72] Webb B, Sali A (2016). Comparative protein structure modeling using MODELLER. Current protocols in protein science.

[73] Melo F, Sánchez R, Sali A (2002). Statistical potentials for fold assessment. Protein science.

[74] Shen MY, Sali A (2006). Statistical potential for assessment and prediction of protein structures. Protein science.

[75] Le Guilloux V, Schmidtke P, Tuffery P (2009). Fpocket: an open source platform for ligand pocket detection. BMC Bioinformatics.

[76] SchuÈttelkopf AW, Van Aalten DM (2004). PRODRG: a tool for high-throughput crystallography of protein-ligand complexes. Acta Crystallogr D Biol Crystallogr.

