# Supplementary material for: Allosteric sodium binding cavity in GPR3: a novel player in modulation of Aβ production
Source: Sci Rep. 2018 Jul 23;8:11102. doi: 10.1038/s41598-018-29475-7 (PMC6056553; doi:10.1038/s41598-018-29475-7)
Supplement: Supplementary file 1 — Supplementary Information [file 41598_2018_29475_MOESM1_ESM.pdf]

Scientific Reports

Supplementary Information

Allosteric sodium binding cavity in GPR3: a novel player in modulation of Aβ production

Stefano Capaldi[a]*, Eda Suku[a]*, Martina Antolini[b], Mattia Di Giacobbe[b], Alejandro Giorgetti[a,c@] and Mario Buffelli[b]

[a] *Department of Biotechnology-University of Verona*
[b] *Department of Neurosciences, Biomedicine and Movement Sciences-University of Verona*
[c]*Computational Biomedicine, Institute for Advanced Simulation IAS-5 and Institute of Neuroscience and Medicine INM-9, Forschungszentrum Jülich, Germany*

**Equal first author contribution*

@Corresponding author
Alejandro Giorgetti, University of Verona, Department of Biotechnology, Strada Le Grazie 15, Verona 37134, Italy, E-mail: alejandro.giorgetti@univr.it

SI 1: *Target-template alignment*. The first sequence indicates our target GPR3 and the second sequence indicates the active template of the Adenosine A_{2a} receptor (PDB code: 5G53). Conserved GPCRs features are indicated with a red circle. Cys166^{TM3} and Cys180^{ECL2} usually form a disulfide bridge in GPCRs, however GPR3 lacks Cys166^{TM3} thus it cannot form this disulfide bridge.

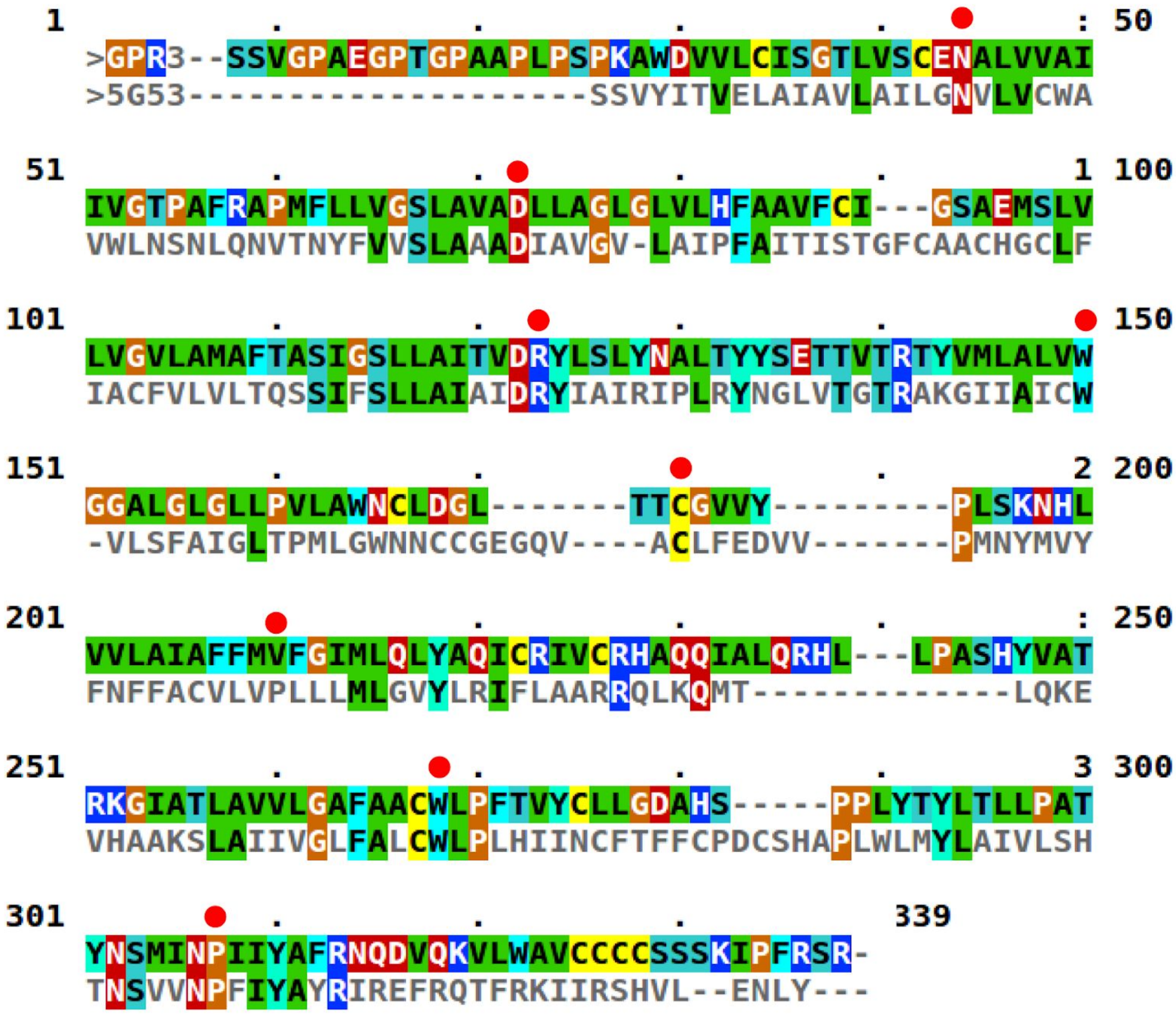

SI 2: *GPR3 residues used as docking restrains for HADDOCK* (UNIPROT code: P46089)

```
92 93 96 97 100 164 165 166 167 174 177 181 234 244 247 263
267
```

SI 3: *List of TAS2R46 residues explicitly indicated as MM in TAS2R46 MM/CG simulations* (UNIPROT code: P46089).

```
1  2  3  4  5  6  7  8  9  10  11  12  13  14  44  45  46  47  48  49  50  51  52  53
54  55  56  57  58  59  60  61  62  63  64  65  66  67  68  69  70  71  72  73  74
75  76  77  78  79  80  81  82  83  122  123  124  125  126  127  128  129  130
131  132  133  134  135  136  137  138  139  140  141  142  143  144  145  146
147  148  149  150  151  152  153  154  155  156  157  158  159  160  161  162
163  164  165  166  167  168  218  219  220  221  222  223  224  225  226  227
228  229  239  231  232  233  234  235  236  237  238  239  240  241  242  243
244  245  246  247  248  249  250  251  252
```

SI 4: *Protein backbone Cα's RMSDs plotted as a function of the simulated time*. The protein reached the equilibration after 350 nanoseconds.

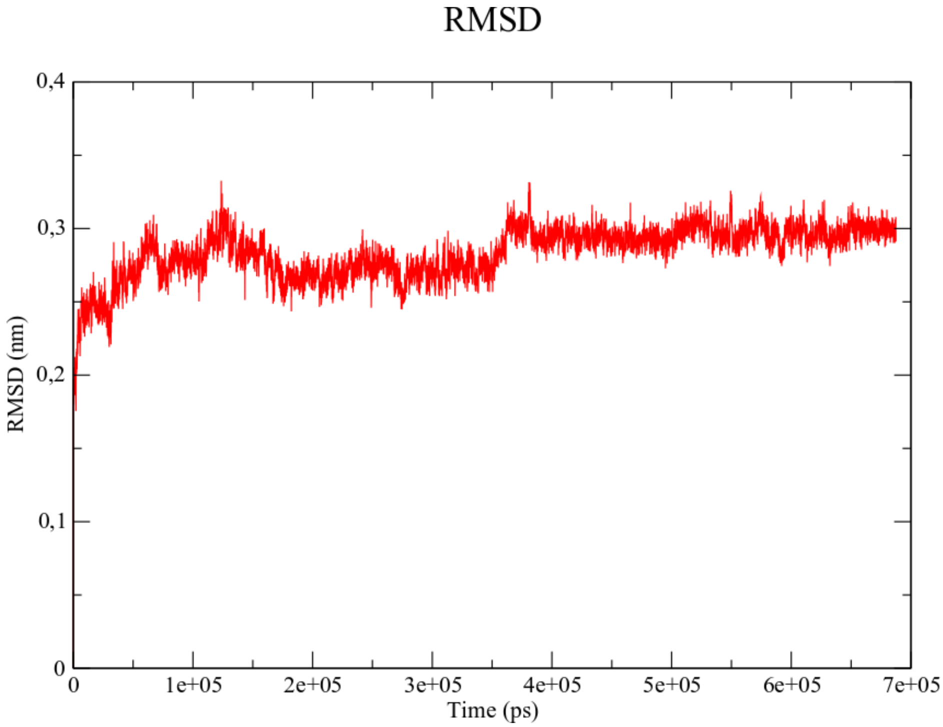

SI 5: *DPI RMSDs plotted as a function of the simulated time*. The ligand reached the equilibration similarly to the GPR3 after 350 nanoseconds.

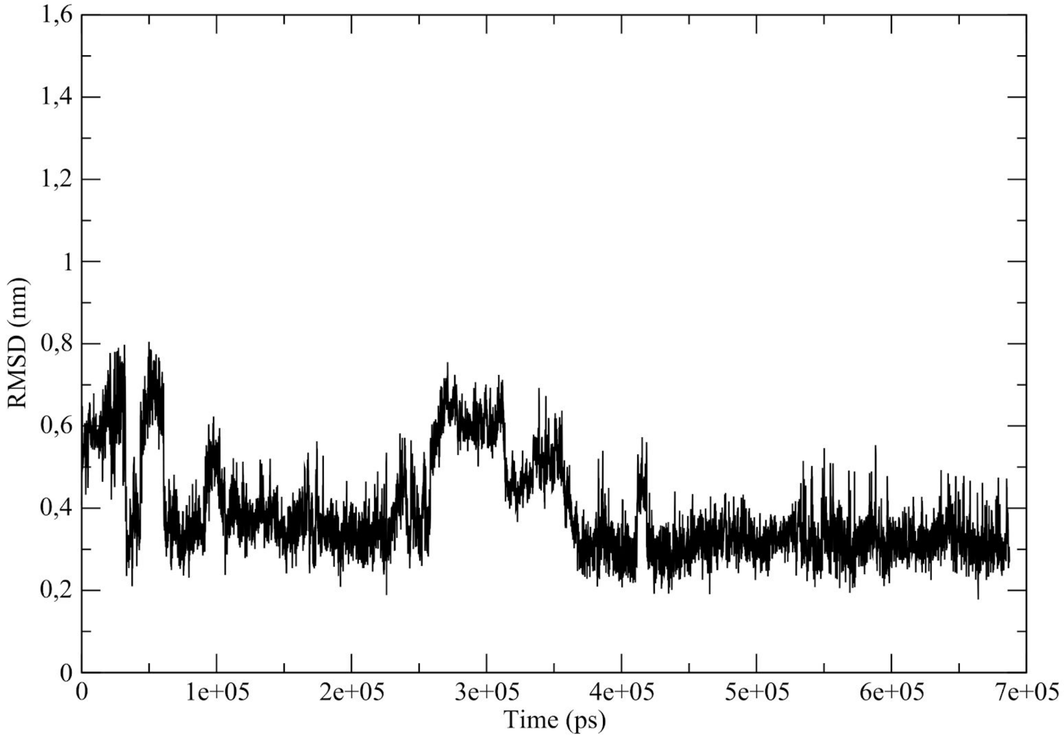

SI 6: *Topology of GPR3 accordingly to GPCRdb*. The snake plot representation of GPR3 was generated with GPCRdb. Violet labels represent mutants that reduced the binding/potency of DPI. In red is shown the mutant that completely abolish the basal activity of the receptor. In orange is shown the part of the receptor inserted in the membrane.

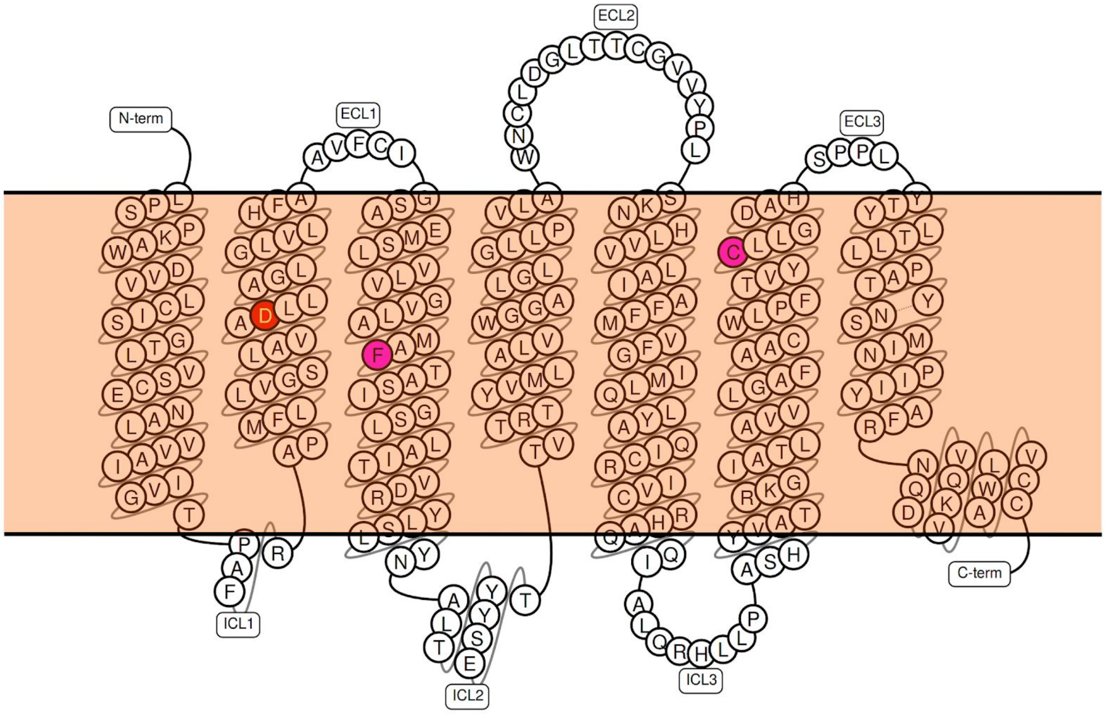

SI 7: Cluster analysis for *in silico* docking between the mutated receptor and the ligand DPI. X-axis is represented by the number of complexes for the most populated cluster and Y-axis is represented by the HADDOCK score. For docking with the mutated receptor a decrease in the number of complexes for the most populated cluster can be noticed.

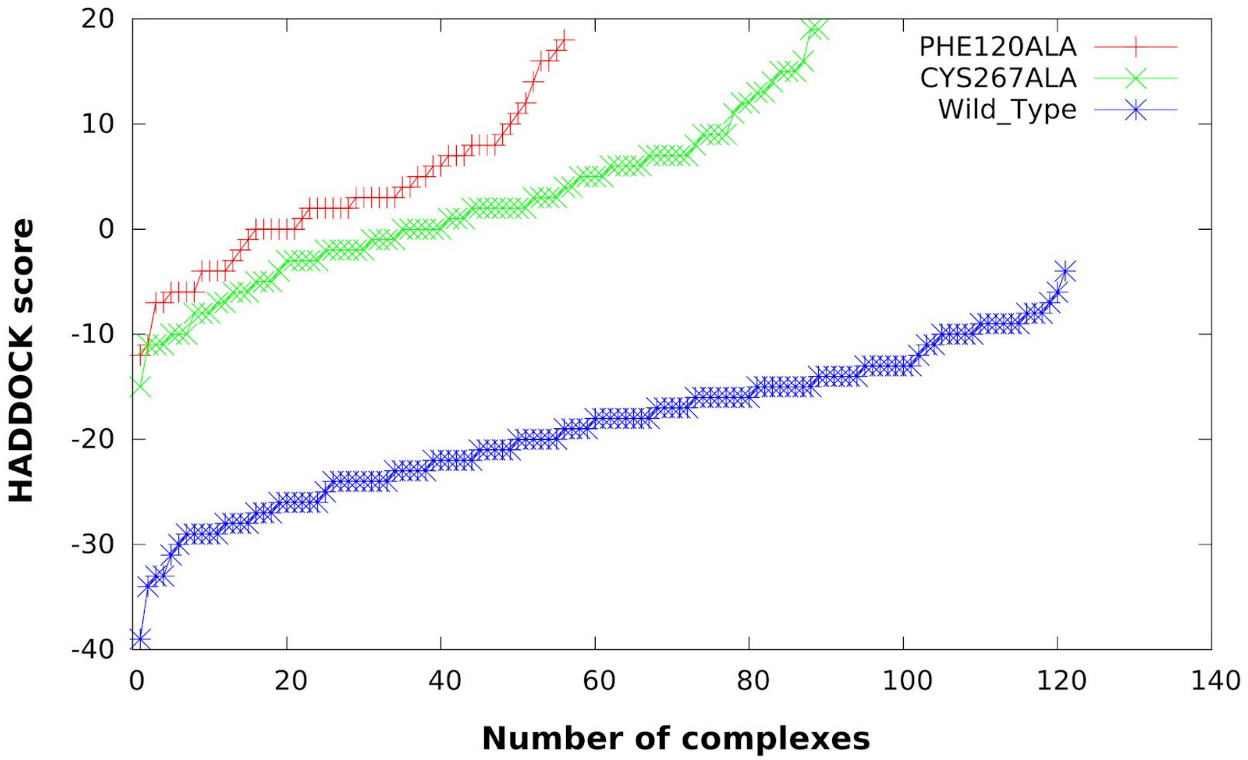

SI 8: Effect of DPI on cell viability. HEK 293 cells were treated with increasing concentrations of DPI (0-50 μM) for 30 minutes (black bars) or 2 hours (red bars) and the cell viability was then assessed by MTT assay.

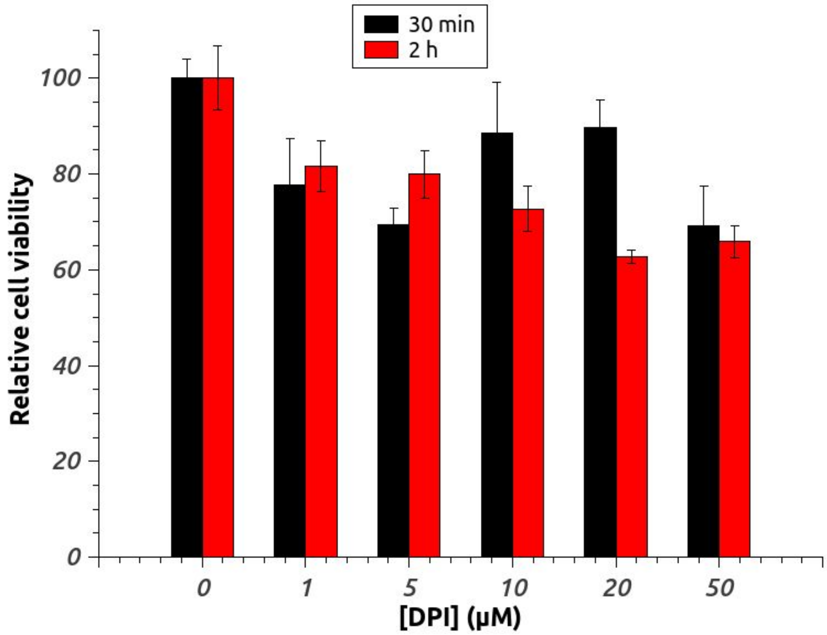

SI 9: DPI parameters used for the simulation with the MM/CG technique. We generated these parameters with the PRODRG server and manually changed the partial charges (underlined in bold). The original charges can be compared with the ACPYPE file in the SI 10. For the sake of clarity only the part of the file regarding the charges is shown.

This file was generated by PRODRG version AA100323.0717

PRODRG written/copyrighted by Daan van Aalten and Alexander Schuettelkopf

Questions/comments to dava@davapc1.bioch.dundee.ac.uk

When using this software in a publication, cite:

A. W. Schuettelkopf and D. M. F. van Aalten (2004).

PRODRG - a tool for high-throughput crystallography of protein-ligand complexes. Acta Crystallogr. D60, 1355--1363.

[moleculetype]

; Name nrexcl

UNK 3

[atoms]

; nr	type	resnr	resid	atom	cgnr	charge	mass
1	CR1	1	UNK	C5	1	**-0.0770**	12.0110
2	HC	1	UNK	H5	1	**0.1700**	1.0080
3	C	1	UNK	C1	1	**-0.0220**	12.0110
4	C	1	UNK	C2	1	**-0.0220**	12.0110
5	CR1	1	UNK	C6	1	**-0.0770**	12.0110
6	HC	1	UNK	H6	1	**0.1700**	1.0080
7	CR1	1	UNK	C9	2	**-0.0740**	12.0110
8	HC	1	UNK	H9	2	**0.1760**	1.0080
9	CR1	1	UNK	C12	2	**-0.0940**	12.0110
10	HC	1	UNK	H12	2	**0.1800**	1.0080
11	CR1	1	UNK	C8	2	**-0.0630**	12.0110
12	HC	1	UNK	H8	2	**0.1790**	1.0080
13	C	1	UNK	C3	2	**0.1139**	12.0110
14	BR	1	UNK	I1	2	**0.0222**	126.9045
15	C	1	UNK	C4	2	**0.1139**	12.0110
16	CR1	1	UNK	C7	2	**-0.0630**	12.0110
17	HC	1	UNK	H7	2	**0.1790**	1.0080
18	CR1	1	UNK	C11	3	**-0.0940**	12.0110
19	HC	1	UNK	H11	3	**0.1800**	1.0080
20	CR1	1	UNK	C10	4	**-0.0740**	12.0110
21	HC	1	UNK	H10	4	**0.1760**	1.0080

SI 10: DPI parameters generated with ACPYPE. We used these parameters to manually changed the charges (underlined in bold) in the parameter file generated with the PRODRG program. The

charges can be compared with the PRODRG file in the SI 9. For the sake of clarity only the part of the file regarding the charges is shown.

```
Remarks dpi3_CNS.top created by acpype (Rev: 401) on Fri Nov  6 14:25:02 2015

set echo=false end

autogenerate angles=True dihedrals=True end

{ atomType  mass }

MASS i_     126.900

MASS cp_     12.010

MASS ca_     12.010

MASS ha_      1.008

RESIdue UNK

GROUP

{ atomName  atomType  Charge }

ATOM I1    TYPE= i_    CHARGE=   0.0222 END

ATOM C1    TYPE= cp_   CHARGE=  -0.0220 END

ATOM C2    TYPE= cp_   CHARGE=  -0.0220 END

ATOM C3    TYPE= ca_   CHARGE=   0.1139 END

ATOM C4    TYPE= ca_   CHARGE=   0.1139 END

ATOM C5    TYPE= ca_   CHARGE=  -0.0770 END

ATOM C6    TYPE= ca_   CHARGE=  -0.0770 END

ATOM C7    TYPE= ca_   CHARGE=  -0.0630 END

ATOM C8    TYPE= ca_   CHARGE=  -0.0630 END

ATOM C9    TYPE= ca_   CHARGE=  -0.0740 END

ATOM C10   TYPE= ca_   CHARGE=  -0.0740 END

ATOM C11   TYPE= ca_   CHARGE=  -0.0940 END

ATOM C12   TYPE= ca_   CHARGE=  -0.0940 END

ATOM H1    TYPE= ha_   CHARGE=   0.1700 END

ATOM H2    TYPE= ha_   CHARGE=   0.1700 END

ATOM H3    TYPE= ha_   CHARGE=   0.1790 END
```

ATOM H4 TYPE= ha_ CHARGE= **0.1790** END

ATOM H5 TYPE= ha_ CHARGE= **0.1760** END

ATOM H6 TYPE= ha_ CHARGE= **0.1760** END

ATOM H7 TYPE= ha_ CHARGE= **0.1800** END

ATOM H8 TYPE= ha_ CHARGE= **0.1800** END

MM/CG technique description:

GPCRs represent the largest membrane protein superfamily, with about 850 members in the human genome and only less than 5% of them have a solved structure. Thus in order to understand the structure of GPCRs and especially GPCRS-agonist complexes we have to rely on computational approaches. Techniques, such as homology modeling along with molecular docking could be a good starting point to model and study the structure of these receptors or study the principal interaction between the ligands and receptors. This could in principle provide insights into agonist/antagonist binding. However, when dealing with very low sequence identity between the target sequence and the available structural templates the construction of reliable alignments is challenging. Moreover, even with a good alignment as a starting point, the construction of the side chains is not accurate and could hinder the correct prediction of docking poses. A way to overcome this is to rely on techniques that explore better the conformation space such as molecular dynamics. All-atom MD has been successfully used in high quality homology models with a sequence identity of more than 60% however, it may provide less satisfying results when the protein structure is built up with a low sequence identity template as for example the case of GPR3. Coarse-grained (CG)-based MD can be used in this case, yet it cannot describe in detail the molecular recognition events between protein and ligand. A way to overcome these limitations is represented by the combination of the two aforementioned techniques. In this context, our group has developed a hybrid "Molecular Mechanics/Coarse-Grained" (MM/CG) method for refinement of GPCRs homology models. In our technique, the system is modeled with two different resolutions. The ligand, the binding site residues and water molecules are treated using an atomistic force field and the rest of the protein is described at a CG level. A coupling scheme is then used to connect the two regions at the boundary. This MM/CG method maintains the atomistic resolution needed to describe correctly the protein–ligand interactions at the binding site, while allowing a larger conformational sampling and a reduced computational cost compared to an all-atom simulation. The presence of the membrane is mimicked by introducing five repulsive walls. Two planar walls coincide with the height of the head groups of the membrane lipids, two hemispheric walls set a limit on the

extracellular and intracellular ends of the protein and the last wall follows the initial shape of the interface between protein and membrane (Leguebe et al., 2012; Marchiori et al., 2013, Sandal et al., 2015, Tarenzi et al., 2017, Suku et al., 2017, Fierro et al., 2017).
